# Cardiovascular Complications of Systemic Therapy in Non-Small-Cell Lung Cancer

**DOI:** 10.3390/jcm9051268

**Published:** 2020-04-27

**Authors:** Magdalena Zaborowska-Szmit, Maciej Krzakowski, Dariusz M. Kowalski, Sebastian Szmit

**Affiliations:** 1 Department of Lung Cancer and Thoracic Tumors, Maria Sklodowska-Curie National Research Institute of Oncology, 02-781 Warsaw, Poland; Magdalena.Zaborowska-Szmit@pib-nio.pl (M.Z.-S.); Maciej.Krzakowski@pib-nio.pl (M.K.); Dariusz.Kowalski@pib-nio.pl (D.M.K.); 2 Department of Pulmonary Circulation, Thromboembolic Diseases and Cardiology, Centre of Postgraduate Medical Education, European Health Centre, 05-400 Otwock, Poland

**Keywords:** cardio-oncology, NSCLC, thromboembolic events, heart failure, acute coronary syndrome, myocarditis, chemotherapy, cardiotoxicity

## Abstract

Cardiovascular diseases may determine therapy outcomes of non-small-cell lung cancer (NSCLC). The evidence for how iatrogenic cardiovascular complications contribute to ceasing anticancer treatment, decreasing the quality of life or even premature death, is unclear. Older patients and smokers are at risk of atherosclerosis and arterial thromboembolic events (TE), such as myocardial infarction or stroke. Venous TE can be observed in up to 15% of NSCLC patients, but the risk increases three to five times in ALK (anaplastic lymphoma kinase)-rearranged NSCLC. ALK inhibitors are associated with electrophysiological disorders. Cytotoxic agents and anti-VEGF inhibitors mainly cause vascular complications, including venous or arterial TE. Cardiac dysfunction and arrhythmias seem to be less frequent. Chemotherapy is often administered in two-drug regimens. Clinical events can be triggered by different mechanisms. Among epidermal growth factor inhibitors, erlotinib and gefitinib can lead to coronary artery events; however, afatinib and osimertinib can be associated with the development of heart failure. During anti-PD1/anti-PDL1 therapy, myocarditis is possible, which must be differentiated from acute coronary syndrome and heart failure. Awareness of all possible cardiovascular complications in NSCLC encourages vigilance in early diagnostics and treatment.

## 1. Introduction

Lung cancer is a highly heterogeneous disease. From a cardiologist’s perspective, it may predispose to thromboembolic complications and often coexists with coronary heart disease and atrial fibrillation [1,2]. From the point of view of an oncologist, it remains one of the main causes of mortality and the prognosis is often comparable or worse than that of advanced heart failure [3]. 

Modern therapy of lung cancer is typically personalized. Apart from chemotherapy, targeted drugs and immunotherapy are used, and indications for particular types of treatment depend on histologic and molecular diagnosis. The functional state of the patient remains of utmost importance. Concomitant diseases, in particular those of the cardiovascular system, may affect the prognosis and choice of treatment.

The efficacy of lung cancer treatment, in particular non-small-cell lung cancer (NSCLC) has increased, but the question remains as to what extent iatrogenic complications force temporary or premature termination of anticancer treatment, decreasing the quality of life or even causing premature death. Publications on thoracic surgery and radiotherapy complications are numerous, but the clinical and prognostic impact of toxicity of systemic therapy in lung cancer is less well researched. 

## 2. NSCLC and Cardiovascular Diseases

Available UK data indicates that in patients with lung cancer, irrespective of age and sex, under long term observation there is increased mortality related to the following cardiovascular diseases: pericarditis (HR = 6.71), venous thromboembolic disease (HR = 5.05), heart failure (HR = 2.14), arrhythmias (HR = 1.97), peripheral arteries disease (HR = 1.93), stroke (HR = 1.78), ischemic heart disease (HR = 1.70) [4]. 

Patients with lung cancer often suffer from concomitant cardiovascular diseases and in the course of antineoplastic treatment may experience various cardiovascular complications (Table 1). Data from an Austrian center in the years 2004–2009 demonstrated that among patients with NSCLC at least one coexisting cardiovascular disease was present in 67.2% of the population [5]. Cardiovascular (CV) complications were documented in 9.5% of patients, and the most common were: conduction abnormalities on electrocardiography (28%), heart failure (19%), myocardial infarction (19%), sudden cardiac death (18%), necessity of coronary revascularization (9%), pericardial effusion (7%). The median interval between diagnosis of NSCLC and cardiovascular complication was 13.9 months. Risk factors for CV complications included: advanced age as well as history of atrial fibrillation, myocardial infarction, and heart disease. However, important cardiologic events did not increase mortality (i.e., did not significantly decrease overall survival; OS). Data from the US [6] based on 95,167 patients with NSCLC showed that cardiovascular diseases may increase mortality in patients with stage I–IIIB disease, while there is no difference in survival in patients with stage IV disease. Poor prognosis was connected with concomitant heart failure, myocardial infarction, and cardiac arrhythmias appearing within the observation time; however, the risk differed depending on the stage of disease and type of treatment. For stage I–IIIB patients coexisting CV diseases increased the risk of mortality by 2.59 (*p* < 0.001) for chemotherapy and by 2.20 (*p* < 0.001) for combined chemo-radiotherapy.

## 3. Anticancer Drugs and Cardiovascular Complications 

### 3.1. Cytotoxic Agents

Cytotoxic agents used in NSCLC seem to cause primarily vascular complications, including venous or arterial thromboembolic events. Sometimes it is difficult to establish the direct mechanism of vasotoxicity connected with the activity of a particular agent. In many cases, there are coexisting risk factors for atherosclerosis, or present cardiovascular diseases, and the drug seems to be an additional factor for triggering clinical symptoms. Also, chemotherapy is often administered in two-drug regimens which makes it difficult to identify the offending agent responsible for a given clinical event. 

#### 3.1.1. Cisplatin

Treatment with cisplatin may be associated with coronary artery disease and its symptoms [7,8]. Patients on cisplatin treatment show significantly higher risk of myocardial infarction [9]. In patients more than 65 years of age cisplatin-containing chemotherapy may produce significantly higher risk of ischemic heart disease or heart failure. The risk of CV toxicity is highest in patients who received chemo-radiotherapy [10]. Chemotherapy based on cisplatin derivatives permanently damages the endothelium and predisposes to obesity, arterial hypertension, and lipid abnormalities, with subsequent increased risk of atherosclerosis, both in coronary and peripheral blood vessels. In young patients with a good long-term prognosis late complications such as arterial hypertension, left ventricle hypertrophy, ischemic heart disease, and infarctions after 10/20 years from termination of treatment, are common [11].

At the cellular level cisplatin triggers platelet activation and aggregation, increases prothrombotic activity, may lead to endothelial dysfunction, increases the concentration of von Willebrand factor, induces hypomagnesemia and thus constriction of blood vessels, and probably also shows antiangiogenic activity [12,13]. Cisplatin may enhance proinflammatory effects and detrimental interactions between endothelial cells, leukocytes, and platelets [14].

Treatment with cisplatin increases the risk of thromboembolic complications [15]. In a retrospective analysis of 932 patients with various types of cancers, the frequency of thromboembolic events related to the use of cisplatin was evaluated between the first dose of chemotherapy and the end of week 4, after the end of chemotherapy [16]. Complications were noted in 18.1% patients including peripheral vein thrombosis (49.7% of events), pulmonary embolism (25.4%), peripheral vein thrombosis and pulmonary embolism (13.6%), arterial thrombosis (8.3%), arterial and venous thrombosis (3.0%). It should be noted that 88% of complications occurred within 100 days from the onset of cisplatin treatment. In unifactorial analysis the following risk factors were identified: gender, age, ethnicity, performance according to Karnofsky scale (KPS), application of drugs stimulating erythropoiesis, presence of venous catheters, site of primary tumor, disease stage, leukocyte count, hemoglobin concentration, results in the Khorana scale. However, in multifactorial analysis the significant factors were only age, KPS, presence of venous catheters, and points in the Khorana scale.

#### 3.1.2. Gemcitabine

Treatment with gemcitabine may lead to vascular complications, including thromboembolic complications [17], in particular thrombotic microangiopathy [18]. During gemcitabine treatment episodes of acute ischemia and even necrosis of distal phalanxes of the limbs were noted [19]. 

Dutch authors analyzed cardiologic safety in patients with NSLC treated with gemcitabine combined with cisplatin, (CG) or epirubicin (EG) [20]. One of the patients receiving EG had myocardial infarction; one patient on CG treatment had atrial fibrillation. A decrease of LVEF >15% was observed only in one patient on CG, and 3 patients on EG. There was no correlation between LVEF decrease and total dose of cisplatin, epirubicin and gemcitabine. However, decrease in LVEF in the whole group correlated with a patient’s age. Patients with a history of myocardial infarction, aortic valve stenosis, or mitral valve insufficiency had a more significant decrease in LVEF. 

In a phase III study of patients with NSCLC, the efficacy and safety of a regimen of cisplatin with gemcitabine was compared with cisplatin monotherapy [21]. Adding gemcitabine to cisplatin led to a higher efficacy of NSCLC treatment. Cisplatin alone evoked grade 3 heart ischemia in 0.4% of patients, while 1.5% of patients treated with cisplatin in combination with gemcitabine were found to have grade 4 heart ischemia. Similarly, arrhythmias of grade 3 occurred only in 0.4% of patients on cisplatin alone, while in patients receiving cisplatin with gemcitabine grade 3 arrhythmia was noted in 1.6% of patients, and grade 4 in 1.2% of patients.

In an interesting phase II study, the efficacy and safety of a regimen containing gemcitabine + pemetrexed + bevacizumab was compared with carboplatin + pemetrexed + bevacizumab [22]. Fatigue and dyspnea were more frequent in patients receiving the regimen with gemcitabine (36% vs.18%), most probably due to induced anemia (22% vs. 7%), while thromboembolic events were as frequent (7%); in the gemcitabine group there was one death from myocardial infarction.

#### 3.1.3. Vinorelbine

Vinca alkaloids may cause autonomic neuropathy [23], coronary artery disease symptoms with altered ECG, including myocardial infarctions [24]. Cardiovascular events associated with vinorelbine use are more common in women [25]. Events characteristic of Prinzmetal angina with typical reversible changes in ECG have also been described, which suggests that ischemia is due to spasms of the coronary vessel.

However, venous complications may be observed. In one report patients with advanced NSCLC (stage IIIB or IV) during the first course of cisplatin and vinorelbine did experience myocardial infarction, as well as pulmonary embolism [26]. In an Italian study, patients treated with vinorelbine and cisplatin more frequently showed venous inflammation than patients treated with gemcytabine and cisplatin [27]. Similarly, Chinese authors observed inflammation of the veins significantly more often in patients receiving vinorelbine with cisplatin, as compared to patients treated with gemcitabine + cisplatin or paclitaxel + cisplatin [28].

#### 3.1.4. Taxanes

Paclitaxel and docetaxel may be used in the treatment of NSCLC (first- or second-line). Symptoms of cardiovascular toxicity differed from asymptomatic bradycardia in ¼ of patients, compared to severe arrhythmias in selected patients. Both atrial and ventricular arrhythmias are present in about 0.5% of patients. Cardiac ischemia was also observed, however, rarely. Also, thrombotic events were noted during paclitaxel treatment [29].

Severe arrhythmias may be acute (during drug influx) or subacute (14 days after administration). In a summary of cardiovascular complications grade 4 and 5 according to the National Cancer Institute (NCI) the frequency of the following abnormalities was determined: supraventricular arrhythmias (tachycardia, atrial fibrillation)—0.24%, ventricular arrhythmias (ventricular tachycardia, ventricular fibrillation)—0.26%, conduction blocks—0.11%, cardiac ischemia—0.29% [30].

Cardiotoxicity of paclitaxel is probably due to the excessive histamine release. Stimulation of histamine receptors in the heart may lead to increased oxygen requirement by cardiomyocytes, contraction of coronary vessels, and altered chronotropic responses. Stimulation of type 1 histamine receptors may cause elongation of atrioventricular conduction, decrease in the activity of Purkinji fibers, and damage of cardiomyocytes. Moreover, paclitaxel may directly affect Purkinji cells as well as the extra cardiac autonomous nervous system.

Cases of sinus bradycardia were documented, as well as atrioventricular conduction blocks, single ventricular contractions, along with ventricular tachycardia caused by paclitaxel [31]. In a large study of about 1000 patients, cardiotoxicity was seen in 14%, and the most common event was asymptomatic bradycardia (76%) [32]. According to the literature the frequency of paclitaxel-related bradycardia is 0.1–31% [33].

#### 3.1.5. Pemetrexed

Serious cardiovascular events such as myocardial infarction, peripheral edema, cardiac arrhythmias, and transient ischemic attack (TAI) were rarely observed [34]. The results of clinical studies show that these events are usually seen when pemetrexed was used in combination with another cytotoxic drug (cisplatin), and they were present in patients with previously diagnosed risk factors of cardiovascular diseases.

### 3.2. Epidermal Growth Factor Receptor Tyrosine Kinase Inhibitors

Epidermal growth factor (EGF) is a potential chemoattractant and mitogen of a smooth muscle cell in the vessels, its expression is increased in the presence of atherosclerotic lesions or vascular remodeling [35]. EGF induces a proliferation of smooth muscle cells and their ability to synthesize collagen fibers. Blocking the EGF by tyrosine kinase inhibitors (TKIs) may make the surface of atherosclerotic plaque more susceptible to damage. EGF receptors (EGFR), apart from affecting the endothelium, can have a more complex effect on coagulation parameters [36].

#### 3.2.1. Erlotinib

Coronary artery events including myocardial infarctions were reported in 2.3% of patients receiving erlotinib 100 mg/day combined with gemcitabine, compared with 1.2% in patients receiving gemcitabine alone for pancreatic cancer [37]. Similarly, thromboembolic events in patients treated with erlotinib + gemcitabine were seen in 3.9%, while the frequency was 1.2% in those receiving gemcitabine alone. Grade 3 or 4 thrombotic complications were present in 11% of patients receiving erlotinib + gemcitabine, as compared with 9% in patients on gemcitabine alone.

Int he BeTa study [38] grade 3/4 thromboembolic complications were seen in one patient treated with erlotinib, while in patients receiving erlotinib with bevacizumab, ten grade 3/4 and two grade 5 events were noted. Similarly, arterial hypertension grade 3/4 was diagnosed in four people (1%) receiving erlotinib and in fifteen (5%) receiving erlotinib along with bevacizumab. In both groups one case of pulmonary embolism was diagnosed.

#### 3.2.2. Gefitinib

The most probable cardiac complication during gefitinib therapy is acute coronary syndrome (ACS). The risk is due to the reactivity of platelets. Observation analysis based on an examination of 20 aspirin-naïve patients showed that gefitinib may activate platelets via a mechanism connected with ADP [39]. It was demonstrated that gefitinib significantly increases the ability of platelets to produce thromboxane A2 (TXA2), thus increasing the prothrombotic ability [40]. According to another hypothesis, gefitinib directly damages the atherosclerotic plaque. Yamaguchi et al. [41] described a case of a 75-year woman with diabetes and arterial hypertension, treated with gefitinib for lung adenocarcinoma; the ACS occurred 2 months after therapy onset. Lynch et al. [42] described a completely different case—recurrent ACS in a 47-year old man without diabetes or hypertension, a nonsmoker. The first episode occurred after 42 months of gefitinib therapy, and coronary angiography revealed a 95% constriction of the left anterior descending artery; angioplasty was done with the insertion of a stent covered with everolimus. Two antiplatelet drugs were recommended (aspirin and clopidogrel) and gefitinib therapy was continued. After a month the patient developed dyspnea and atypical chest pain. Upon coronary angiography no significant changes were found, but an analysis of the platelet function was performed. Due to increased concentration of thromboxane, despite aspirin therapy, the dose of clopidogrel increased from 75 mg to 150 mg per day. Following six weeks of gefitinib therapy another ACS occurred, and this time angiography revealed 95% constriction of the left circumflex artery. Another angioplasty was performed with the insertion of an everolimus-covered stent. Gefitinib was discontinued and within the following two years no coronary events were noted. In this patient an increased concentration of thromboxane was found despite aspirin and clopidogrel administration.

#### 3.2.3. Afatinib

Afatinibis a non-reversible inhibitor of the ErbB family of receptors(epidermal growth factor receptor—EGFR/ErbB1, human epidermal growth factor receptor 2—HER2/ErbB2, HER4-ErbB4) [43]. Inhibition of the HER2 receptor raised concerns about the cardiological safety of afatinib. Analysis of data from clinical studies, LUX-Lung 3 (229 patients treated with afatinib), LUX-Lung 1 (390 patients treated with afatinib) and 49 other studies (3865 patients treated with afatinib), was designed to determine the risk of heart failure [44]. It appeared that the occurrence of events in clinical randomized studies for 100 patient-years (events/100 patient-years) was similar for afatinib and a placebo (2.40 vs. 2.23), as well as for afatinib and chemotherapy (2.28 vs. 2.92); similar frequency was seen in other studies without randomization (2.88). Significant decrease in LVEF was more frequent in the course of chemotherapy compared with afatinib (13.3% vs. 6.3%; *p* = 0.267). However, when afatinib was compared with a placebo, the frequency of significant LVEF decrease was similar (4.1% vs. 4.6%).

#### 3.2.4. Osimertinib

There are comparisons of Osimertinib cardiotoxicity versus other EGFR-TKIs (erlotinib, afatinib, gefitinib) [45]. Retrospective data from 2016–2018 showed that treatment with Osimertinib significantly increases the risk of: QT prolongation, heart failure, and atrial fibrillation. Among the 8450 reported adverse events related to EGFR-TKI 2454 were due to osimertinib, whereas 5836 were associated with other EGFR-TKIs (erlotinib, afatinib, gefitinib). The median time interval until heart failure occurrence was 29 and 23 days, representing significant prolongation of the QT interval.

The problem of prolonged QT was reported in a phase III study aimed at demonstrating the superiority of osimertinib over chemotherapy with pemetrexed and cisplatin/carboplatin [46]. Grade 1 or 2 complications were observed in 4% of patients, but grade 3 only in one out of 273 patients from the osimertinib group. In this study, one patient, treated with osimertinib, experienced ischemic cerebral stroke. Cardiotoxicity, defined as decrease of LVEF by at least 10% and below 50%, was noted in 5% of cases and the median time before complications was 5.5 months.

In clinical studies confirming the superior efficacy of Osimertinib over standard EGFR-TKI (gefitinib or erlotinib), prolonged QT was reported in 10% of patients on osimertinib (2% grade 3, below 1% grade 4), compared with 4% receiving gefitinib/erlotinib (1% grade 3) [47]. In the same study LVEF decrease was noted in 3% of patients on osimertinib and 1% of those receiving gefitinib or erlotinib.

Prolonged QT, defined as grade 3, represents a significant clinical problem as it requires therapy discontinuation due to excessive risk of life threatening ventricular tachycardia. In a meta-analysis the percentage of QT prolongation grade 3 cases for Osimertinib therapy was determined to be 2% [48]. Unfortunately, discontinuation of anti-EGFR therapy may result in rapid progression of cancer [49]. Thus, any decision to with drawosimertinib should be carefully considered, in particular in metastatic disease [50].

The mechanism behind QT prolongation seems to be connected with inhibition of the PI3K pathway, which was demonstrated on animal models [51]. Elongation of QT over 500 msis connected with a significantly higher (2.5 times higher compared to persons with normal QT) risk of serious cardiac arrhythmias. Additional risk factors include electrolyte abnormalities (hypokalaemia), bradycardia, and administration of other drugs which may prolong QT. Family history of sudden cardiac death or congenital long QT syndrome is another factor to consider.

Initial descriptions of heart failure during osimertinib treatment shows that there may be no abnormalities in QT on an ECG. In addition, generalized hypokinesis of the left ventricle and low LVEF, was observed, as well as low volume pericardial effusion, no significant changes on magnetic resonance imaging. In biopsy lymphocyte infiltration and features of edema were found [52]. Another case showed dyspnea, face and limbs edema, and bilateral pleural effusion, along with interstitial lung edema on computed tomography [53]. Further diagnosis excluded any inflammatory cause. The authors explained the development of acute heart failure by the fact that Osimertinib inhibits the HER2 receptor [54].

### 3.3. ALK Inhibitors

About 8% to 15% of patients with NSCLC may experience venous thromboembolic events (VTE) and certainly chemotherapy increases this risk (as described above). Surprisingly, in a specific molecular subtype of NSCLC such as ALK (anaplastic lymphoma kinase)—rearranged, the incidence of VTE can also be three to five times higher. It was confirmed that 36% of the patients who had VTE, were younger (*p* = 0.04), had a worse Eastern Cooperative Oncology Group performance status (*p* = 0.04), and higher mortality risk (HR = 5.71, *p* = 0.01) [55]. A phase II study with the first representatives of targeted therapy (crizotinib) in ROS1-rearranged NSCLC, demonstrated that VTE with different localizations in the venous system can occur at NSCLC diagnosis (32.1%) or progression (35.7%), as well as during chemotherapy (17.8%) or crizotinib treatment (14.2%) [56]. To date nobody has confirmed the relationship between a risk of VTE and the activity of ALK inhibitors.

#### 3.3.1. Crizotinib

Crizotinibis an inhibitor of ALK and ROS1. In clinical practice two types of ECG complications are observed: bradycardia (heart rate <60 beats per minute) and QT prolongation [57]. Bradycardia is caused by the detrimental effect of crizotinib on the activation of HCN4 (hyperpolarization-activated cyclic nucleotide-gated channel 4) in cardiomyocytes [58]. Another cause is a decrease in testosterone concentration [59]. There are other risk factors for bradycardia including, advanced age, poor functional state, and a longer time of crizotinib therapy (bradycardia may appear after many weeks of treatment) [60]. In clinical phase III studies (PROFILE 1007, 1014) the frequency of bradycardia was just over 10% and in the majority of cases it was mild, asymptomatic, rarely connected with fainting, hypotension, or dizziness [61,62]. However, prolongation of QTcF (Fridericia’s formula) ≥500 ms was observed in 2.1% of patients, and relative prolongation by ≥60 ms in 5% of cases.

#### 3.3.2. Ceritinib

Ceritinibis a selective and strong inhibitor of ALK. ECG abnormalities include bradycardia in 1.9% of patients and in 6.5% events connected with prolongation of QTC: grade 3 and 4—0.8% patients, dose reduction or temporary discontinuation of treatment in 1% and termination of treatment in 0.2%. A specific cardiovascular complication of ceritinib therapy seems to be pericarditis or pericardial effusion, observed in 5.9% of patients [63].

#### 3.3.3. Alectinib

Alectinibis an inhibitor of tyrosine kinases ALK and RET. In a phase II study the correlation between alectinib concentration and changes in QTcF, was analyzed, as well as the effect of alectinib on heart rates, PR intervals and the duration of QRC. The study did not reveal any clinically significant effect of alectinib on ECG, aside from asymptomatic decrease in the heart rates by 11–13 beats per minute [64].

#### 3.3.4. Brigatinib

Brigatinib is an inhibitor of ALK and EGFR. In early phase studies the most common cardiological side effect, grade 3–4, requiring immediate treatment was arterial hypertension (5%) [65].

### 3.4. VEGF Inhibitors

A complication typical for the whole group of drugs blocking VEGF-dependent pathways is the development of arterial hypertension. The mechanism behind this is thought to be a decrease in nitrous oxide synthase and an increase in the activity of PAI-1 (plasminogen activatorinhibitor-1). Some authors suggest a mechanism connected with the activation of the renin-angiotensin-aldosterone system. A mechanism related to lower sodium ions kidney excretion, is also probable. Inhibition of VEGF-dependent pathways leads to the destruction of microcirculation, reduces endothelial growth and possibility inhibits the development of capillary vessels. Decreased density of microcirculation has also been described (the so-called rarefaction). Clinical consequences include increased peripheral vascular resistance and increased arterial blood pressure [66]. However, initially clinical studies in oncology interpreted 150/100 mmHg as a threshold for arterial hypertension, and not 140/90 mmHg, as in cardiology. This has changed with the introduction of the Common Terminology Criteria for Adverse Events v4.0.

#### 3.4.1. Bevacizumab

The most common cardiovascular complication of bevacizumab therapy is arterial hypertension. Initial data suggests a frequency of 35% in treated patients, out of which 20% were cases diagnosed de novo, while in 80% of cases, increased arterial blood pressure was seen in patients with prior hypertension (exacerbation of pre-existing hypertension) [67]. It should be noted that in patients with NSCLC treated with bevacizumab, grade 3 or 4 arterial hypertension occurred with a frequency of 5.7% (dose 15 mg/kg) [68] which is less than in other neoplasms: for example in renal cancer—20.5% (dose 10 mg/kg) [69] or breast cancer—17.9% (dose 15 mg/kg) [70].

In a phase II study, the efficacy of carboplatin alone and paclitaxel with a low dose (7.5 mg/kg) or high dose (15 mg/kg) of bevacizumab was compared [71]. Arterial hypertension was diagnosed in 5 out of 32 (15.6%) of those on a low dose of bevacuizumab, 6 out of 35 (17.1%) who received a high dose of bevacizumab, and in 1 out of 32 (3%) on chemotherapy alone. In this study, arterial hypertension did not lead to discontinuation of therapy.

In an ECOG study 4599 patients with non-squamous NSCLC were randomized for treatment with carboplatin and paclitaxel, combined with bevacizumab or not (15 mg/kg every 21 days). The prognosis of patients with iatrogenic arterial hypertension diagnosed by the end of first cycle (criterion >150/100 mmHg or increase of diastolic pressure by 20 mmHg) was compared with the prognosis of patients without hypertension [72]. Patients with iatrogenic hypertension on carboplatin + paclitaxel + bevacizumab compared with normotensive subjects on the same chemotherapy showed significantly longer OS (HR = 0.60; *p* = 0.001). Progression-free survival was also significantly longer (HR = 0.54; *p* < 0.0001). A comparison between patients on carboplatin + paclitaxel + bevacizumab without hypertension, with patients on chemotherapy alone, showed better OS in the first group (HR = 0.86; *p* = 0.05), as well as better progression-free survival (HR = 0.72; *p* < 0.0001). These results suggest that hypertension induced by bevacizumab may be regarded as a positive predictive factor. Because the degree of arterial hypertension can correlate with the dose of bevacizumab [73], some authors suggest beginning bevacizumab therapy with a low dose and then gradually increasing it. Others advocate thorough monitoring of blood pressure along with the rapid onset of hypotensive therapy. Angiotensin converting enzyme inhibitors and calcium channel blockers seem to be effective drugs [74]. Antihypertensive treatment should involve a combination of drugs with various mechanisms of action. Angiotensin converting enzyme inhibitors seem most effective due to beneficial effects on microcirculation and endothelial function [75]. Routine reduction of bevacizumab dose is not recommended as a hypotensive approach, because it may impair the efficacy of antineoplastic treatment.

Another cardiovascular complication of bevacizumab therapy is cardiac dysfunction [76]. The pathomechanism of heart muscle damage is probably due to uncontrolled iatrogenic arterial hypertension and the inhibition of VEGF—dependent pathways. Animal studies revealed that iatrogenic inhibition of VEGF leads to an increase of afterload, decrease of capillary vessels in myocardium, global systolic dysfunction, myocardial fibrosis, and decompensation of circulation [77]. From the clinical point of view the essential observation is that cardiac dysfunction triggered by bevacizumab can potentially resolve spontaneously [78]. In their meta-analysis Choueiri et al. [79] analyzed the data of 3784 patients with breast cancer from 1966–2010, including 2366 patients on bevacizumab. The frequency of iatrogenic symptomatic cardiac dysfunction induced by bevacizumab was 1.6%. Relative risk was 4.74 as compared with patients on chemotherapy without bevacizumab (*p* = 0.001). There was no correlation with the dose of bevacizumab or type of additional cytotoxic agents (taxanes vs. capecitabine vs. anthracyclines, *p* = 0.75). This meta-analysis has limitations, as there are no data on asymptomatic cardiac dysfunction caused by bevacizumab, iatrogenic arterial hypertension, and thromboembolic events, nor on real risk factors of heart failure, nor on the effect of iatrogenic cardiac dysfunction on overall survival. Another problem is the risk of heart failure associated with the advanced age of patients [80].

Thromboembolic events are a serious vascular complication of bevacizumab therapy. In a pooled analysis of 1745 patients with NSCLC or other metastatic carcinoma from 5 randomized studies, the overall occurrence of these events was 3.8% [81]. The frequency of myocardial infarctions type 1 (resulting from thrombosis/embolus in a coronary artery) was only 1.5%. These complications may appear at any time during therapy; however, available data show that it usually occurs around the third month of treatment. The risk does not seem to be related to a single dose nor a cumulative dose. Being older than 65 years of age and a history of thromboembolic events are the strongest risk factors. The mechanism behind such complications has not been explained; however, it is probably due to the inhibition of VEGF-dependent pathways, as VEGF stimulates the proliferation of endothelial cells, enhances survival of endothelial cells and the integrity of blood vessels [82]. Inhibitors of VEGF such as bevacizumab decrease the regenerative ability of endothelial cells when damaged, and leads to endothelial dysfunction. They also expose collagen from the subendothelial layer, which activates the tissue factor and increases the prothrombotic activity. Additionally, the inhibition of VEGF leads to a decrease in nitric oxide and prostacyclins (vasodilatants), while inducting the erythropoietin production it increases hematocrit and blood viscosity. Interestingly, in the analysis with NSCLC patients in contrast to other types of cancer disease, treatment with bevacizumab did not increase significantly the risk of arterial (*p* = 0.16) and venous adverse events (*p* = 0.64) [83].

Patients with severe thromboembolic events should discontinue bevacizumab therapy. There is no data on the safety of subsequent bevacizumab administration after successful antithrombotic treatment. No evidence of increased risk of bleeding was found in patients on anticoagulants who receive bevacizumab [84].

#### 3.4.2. Nintedanib

Nintedanib inhibits three proangiogenic pathways connected with the activity of VEGFR (vascular endothelial growth factor receptors), PDGFR (platelet-derived growth factor receptors), and FGFR (fibroblast growth factor receptors). Apart from the oncological setting, nintedanibis used in idiopathic pulmonary fibrosis. The cardiovascular safety of nintedanib was investigated in clinical studies [85]—serious cardiovascular events were as frequent in nintedanib and in a placebo group, both in patients with and without risk factors for atherosclerosis. The frequency of myocardial infarction increased significantly (3.03 per 100 patient-years) in persons with risk factors. Benefits from nintedanib in the second-line treatment of patients with NSCLC were documented in randomized clinical studies, LUME-Lung 1 with docetaxel [86], and LUME-Lung 2 with pemetrexed [87]. Additional analysis showed that arterial hypertension and thromboembolic complications were rare [88]. Thus, unlike other antiangiogenic TKI, nintedanib less frequently produced arterial hypertension and cardiovascular events. Safety evaluation in the LUME-Lung1 study revealed a higher frequency of some adverse events in docetaxel + nintedanib arm compared with docetaxel alone, including: arterial hypertension (3.5% vs. 0.9%), bleeding (14.1% vs. 11.6%), thromboembolic events of all degrees (5.1% vs. 4.6%) [89].

### 3.5. Immune Checkpoint Inhibitors (ICIs)

Various adverse events may appear duringanti-PD1/anti-PDL1 treatment as a result of autoimmune reactions and as complications independent from the activity of the immune system [90]. Large clinical studies on the efficacy of pembrolizumab, nivolumab and atezolizumabin NSCLC revealed a low risk of cardiovascular events (<1%). In clinical studies where pembrolizumab was given in NSCLC in combination with chemotherapy, the risk of cardiac complications did not increase significantly [91,92]. However, when durvalumab was administered after radiochemotherapy in patients with stage III NSCLC, cardiovascular events were seen in 4.4% of cases [93]. Current FDA data on 21,664 patients from 59 clinical studies suggests that ICI is connected with a higher frequency of myocarditis, vasculitis, ischemic episodes, arrhythmias, and pericardium diseases [94].

The majority of immune complications occur very early, the median time to their occurrence is a maximum of 65 days [95]. Based on the results of 21 early phase studies, serious complications of immunotherapy (including cardiovascular at 2%) were several times more frequent during the first four weeks than in subsequent weeks [96]. Late cardiovascular complications have also been described, but according to French data those occurring later than 90 days from immunotherapy onset are typically cases of heart failure with left ventricular systolic dysfunction [97].

In one of the retrospective analyses the risk of serious cardiovascular events in NSCLC such as cardiovascular death, myocardial infarction, stroke, or cardiac insufficiency requiring hospitalization, was evaluated with and without immunotherapy. It revealed the frequency of these complications to be in the range of 13% (as compared with 10% in patients receiving other types of therapy) [98]. Clinical characteristics of the group were not related to the risk of serious cardiovascular events. There was no strict algorithm for evaluation of troponin biomarkers and NTproBNP; however, increase of these biomarker concentrations during immunotherapy correlated with the risk of significant cardiovascular events—for troponin it was defined as TnI > 0.01 ng/mL (HR = 7.27; *p* < 0.001) and BNP > 100 pg/mL (HR = 2.65; *p* = 0.047). In another multicenter study (including patients with NSCLC) increased BNP correlated with the occurrence of cardiomyopathy or arrhythmias during immunotherapy [99]. This finding was confirmed in another study—increased troponin level was a predictor of myocarditis and MACE (major adverse cardiac events), but vascular events were not analyzed [100]. Increased troponin levels in patients with NSCLC should be interpreted with care as it may represent sequelae of previous radiotherapy, chronic heart ischemia in elderly patients with atherosclerosis, or may be caused by non-cardiological factors such as renal dysfunction [101].

One of retrospective observation studies revealed that patients exposed to immune therapy had increased risk of acute vascular complications during first six months of treatment, the risk being twice as high [102]. Atherosclerosis is associated with inflammatory processes and thus immunotherapy may alter its course. In the quoted study from 2015–2018 vascular events during immunotherapy for NSCLC were noted, and among the 1215 patients 2.6% of them experienced such events, while in lung adenocarcinoma it was 5.2%. Lung adenocarcinoma, history of vascular events and dyslipidemia were significant risk factors for vascular complications during immunotherapy. Interestingly, in lung adenocarcinoma the frequency of vascular complications was similar for immunotherapy and chemotherapy. However, vascular complications were correlated with a worse prognosis (shorter survival).

The mechanism and clinical significance of pericarditis during immunotherapy is difficult to explain. It seems to be most frequent during nivolumab therapy [103], and may be recurrent [104,105]. The study aimed at determining the frequency of hemodynamically significant pericardial effusion requiring pericardiocentesis, found in a group of 3966 patients on ICIs that the procedure was seldom indicated: during nivolumab treatment—0.61%, pembrolizumab—0.19%andatezolizumab—0.32%. The survival of patients after pericardiocentesis did not differ between patients on ICIs and those receiving other anticancer drugs [106].

Meta-analyses on the safety of ICIs are now available. In a meta-analysis from 2017 [107] including 22 clinical studies in NSCLC the frequency of cardiopulmonary insufficiency was 1.0%, cardiac insufficiency—2.0%, myocardial infarction—1.0%, stroke—2.0%. A meta-analysis from 2019 including over 20,000 patients, with various neoplasms on ICIs, showed the risk of cardiovascular complications at 9.8%, mainly heart failure/cardiomyopathy and myocardial infarction [108].

A summary of FDA data was presented (the FDA Adverse Events Reports System, FAERS) for the years 2017–2018 [109]. This review was dedicated to all neoplasms and all available ICIs (pembrolizumab, nivolumab, atezolizumab, avelumab, durvalumab, ipilimumab)—it was concluded that many events occurred in patients with lung cancer. In total there were 36,848 reported toxic effects of ICIs and cardiovascular events amounted to 6.2% and included premature cardiac deaths. Clinical diagnoses comprised: myocarditis (15%), atrial fibrillation (13%), pericardial diseases, including effusion (13%), heart failure (17%) and acute coronary syndromes (19%). The highest mortality was seen for myocarditis (50%), acute coronary syndromes (43%) and heart failure (40%), slightly less in atrial fibrillation (22%) and pericardial complications (15%).

## 4. Early Diagnosis of Cardiovascular Toxicity in NSCLC

Treatment of patients with NSCLC may be associated with a risk of serious cardiovascular events. Cardiac dysfunction and arrhythmias seem to be less frequent. During immunotherapy a specific new complication is possible—autoimmune myocarditis, which must be differentiated from acute coronary syndrome and heart failure of another origin [110]. In pericardial effusion it is crucial to exclude neoplastic disease progression, as it will determine the management [111].

Early recognition of all possible iatrogenic cardiovascular events seems to be crucial for optimal treatment (Table 2). Only in this way can premature cardiovascular deaths be avoided. Paradoxically, the simplest diagnosis seems to be in a case of iatrogenic hypertension. Home measurements should be preferred because of the avoidance of the white coat effect, and stress experienced by cancer patients in a hospital or doctor’s office. The basis for recognizing arrhythmias is ECG. However, diagnosis of asymptomatic or very rare arrhythmias (e.g., once a week or less) can be troublesome because it requires long-term ECG monitoring. In current daily practice, diagnosis of acute venous or arterial vascular adverse events should be based on the occurrence of clinical symptoms. However, we would like to have markers that would allow us to recognize these acute conditions before the onset of acute symptoms. If we want to recognize myocarditis, frequent assessment of troponin and ECG appears to be the most appropriate method. Finally, for early diagnosis of cardiac dysfunction, echocardiographic monitoring and, above all, strain assessment should be proposed. However, one should be aware that many patients with NSCLC appear after thoracic surgery and radiation therapy. Thus, the quality of echocardiographic imaging may be decreased and cardiac magnetic resonance imaging (MRI) and its strain assessment become an important alternative.

## 5. Conclusions

The diversity of cardiovascular complications in NSCLC makes it difficult to plan formal cardiology monitoring. The relatively low frequency of such events instills doubt as to the cost-effectiveness of such monitoring. However, knowledge of these complications leads to vigilance in early diagnostics and treatment, as only such approaches may enhance the benefit resulting from applied anticancer therapy. Clinicians should be watchful for mainly vascular complications and thus preventive strategies should involve optimal control of all potential risk factors.

Unfortunately, current cardio-oncology standards do not concentrate adequately on patients with NSCLC who receive systemic therapy [112,113]. The majority of patients with NSCLC are elderly and were smokers in the past. Keeping in mind that most of them suffer from concomitant heart disease, it is crucial to plan further studies with prognostic potential for this specific population of patients.

## Figures and Tables

**Table 1:** Relationships between cardiovascular events and activity of drugs used in NSCLC.

		Cardiac Toxic Effects			Vascular Toxic Effects		
		Heart Failure/Cardiac Dysfunction	Atrial Fibrillation/ECG Changes	Myocarditis/Pericarditis	Venous Thromboembolism	Acute Coronary Events	Hypertension
Cytostatic agents	Cisplatin	↑ in elderly	↑		↑↑↑	↑↑↑	↑ as late effect
	Gemcitabine		↑↑↑			↑↑↑	
	Vinorelbine				↑ combined with cisplatin	↑↑↑	
	Taxanes		↑↑↑			↑↑↑	
	Pemetrexed					↑ in combined chemotherapy	
EGFR inhibitors	Erlotinib				↑↑↑	↑↑↑	
	Gefitinib					↑	
	Afatinib	↑					
	Osimertinib	↑↑↑	↑↑↑				
ALK inhibitors	Crizotinib		↑↑↑				
	Ceritinib		↑↑↑	↑			
	Alectinib		↑				
	Brigatinib						↑
VEGF inhibitors	Bevacizumab	↑			↑↑↑	↑↑↑	↑↑↑
	Nintedanib					↑↑↑	↑↑↑
Immune checkpoint inhibitors	Pembrolizumab	↑ differential diagnosis of reasons is needed		↑↑↑		↑↑↑	
	Nivolumab						
	Atezolizumab						
	Durvalumab						

Legend: ↑↑↑-clearly confirmed effect; ↑-observed effect in selected patients.

**Table 2:** Cardiovascular events induced by NSCLC therapy and possible strategy of early diagnosis.

Cardiovascular Event	Drugs Used in NSCLC and Associated with Risk of Cardiovascular Events	Possible Strategy of Early Diagnosis
Heart failure/cardiac dysfunction	Chemotherapy: Cisplatin EGFR inhibitors: Afatinib, Osimertinib	Routine echocardiography with repeat left ventricular ejection fraction (LVEF) measurements as well as global longitudinal strain (GLS) evaluations.
	VEGF inhibitors: Bevacizumab Immune checkpoint inhibitors	LVEF and strain measurements by magnetic resonance in patients with reduced quality of echocardiographic imaging.
Atrial fibrillation/ECG changes	Chemotherapy: Gemcitabine, Taxanes EGFR inhibitors: Osimertinib ALK inhibitors: Crizotinib, Ceritinib Immune checkpoint inhibitors	Routine ECG at each visit Longterm ECG monitoring in patients with suspected asymptomatic or rare arrhythmias
Myocarditis	ALK inhibitors: Ceritinib	Routine ECG and troponin evaluation at each visit
	Immune checkpoint inhibitors	Cardiac magnetic resonance and myocardial biopsy at symptoms occurrence
Venous thromboembolism	Chemotherapy: Cisplatin, Vinorelbine EGFR inhibitors: Erlotinib VEGF inhibitors: Bevacizumab	Computed tomographic pulmonary angiography and/or compression ultrasonography at symptoms occurrence
Acute coronary events	Chemotherapy: Cisplatin, Gemcitabine, Vinorelbine, Taxanes	Invasive coronary angiography as standard at acute symptoms occurrence
	EGFR inhibitors: Erlotinib, Gefitinib	
	VEGF inhibitors: Bevacizumab, Nintedanib	Coronary computed tomography angiography as alternative for selected patients
	Immune checkpoint inhibitors	
Arterial hypertension	Chemotherapy: Cisplatin ALK inhibitors: Brigatinib	Home blood pressure measurements Routine blood pressure control at each visit
	VEGF inhibitors: Bevacizumab, Nintedanib	
